# Optimization of Efficient Neuron Models With Realistic Firing Dynamics. The Case of the Cerebellar Granule Cell

**DOI:** 10.3389/fncel.2020.00161

**Published:** 2020-07-14

**Authors:** Milagros Marín, María José Sáez-Lara, Eduardo Ros, Jesús A. Garrido

**Affiliations:** ^1^Department of Computer Architecture and Technology—CITIC, University of Granada, Granada, Spain; ^2^Department of Biochemistry and Molecular Biology I, University of Granada, Granada, Spain

**Keywords:** neuron model, granule cell, cerebellum, model simplification, spiking resonance, point neuron, adaptive exponential integrate-and-fire

## Abstract

Biologically relevant large-scale computational models currently represent one of the main methods in neuroscience for studying information processing primitives of brain areas. However, biologically realistic neuron models tend to be computationally heavy and thus prevent these models from being part of brain-area models including thousands or even millions of neurons. The cerebellar input layer represents a canonical example of large scale networks. In particular, the cerebellar granule cells, the most numerous cells in the whole mammalian brain, have been proposed as playing a pivotal role in the creation of somato-sensorial information representations. Enhanced burst frequency (spiking resonance) in the granule cells has been proposed as facilitating the input signal transmission at the theta-frequency band (4–12 Hz), but the functional role of this cell feature in the operation of the granular layer remains largely unclear. This study aims to develop a methodological pipeline for creating neuron models that maintain biological realism and computational efficiency whilst capturing essential aspects of single-neuron processing. Therefore, we selected a light computational neuron model template (the adaptive-exponential integrate-and-fire model), whose parameters were progressively refined using an automatic parameter tuning with evolutionary algorithms (EAs). The resulting point-neuron models are suitable for reproducing the main firing properties of a realistic granule cell from electrophysiological measurements, including the spiking resonance at the theta-frequency band, repetitive firing according to a specified intensity-frequency (I-F) curve and delayed firing under current-pulse stimulation. Interestingly, the proposed model also reproduced some other emergent properties (namely, silent at rest, rheobase and negligible adaptation under depolarizing currents) even though these properties were not set in the EA as a target in the fitness function (FF), proving that these features are compatible even in computationally simple models. The proposed methodology represents a valuable tool for adjusting AdEx models according to a FF defined in the spiking regime and based on biological data. These models are appropriate for future research of the functional implication of bursting resonance at the theta band in large-scale granular layer network models.

## Introduction

Neuronal populations in the brain reflect complex synchronized temporal patterns typically modulated by coherent oscillations (Buzsáki, 2006). This oscillatory behavior is usually evidenced by the study of resonance as the preferred frequency in response to oscillatory inputs (Hutcheon and Yarom, 2000). In particular, one of the brain centers where resonance has received more attention is the cerebellum (Dugué et al., 2009; D’Angelo et al., 2009, 2011; Gandolfi et al., 2013). The cerebellum is thought to generate low-frequency (5–30 Hz) and higher-frequency activity rhythms, depending on the circuit sections or the neurons involved (D’Angelo et al., 2009; Dugué et al., 2009). Previous findings suggest that theta-frequency activity (around 4–10 Hz in rodents) contributes to signal integration in the cerebellum (Gandolfi et al., 2013), but its function for overall cerebellar information processing remains elusive.

The cerebellar granular layer (GrL) represents one of the main inputs to the cerebellar cortex and low-frequency rhythms at this layer is fundamental for motor control, learning, and sleep (Buzsáki, 2006; D’Angelo et al., 2009; Wang et al., 2019). Most studies have focused on subthreshold (membrane potential oscillations) resonance. In particular, *in* *vivo* studies of cerebellar GrL evidenced theta-frequency resonance at 7 Hz in rats (Hartmann and Bower, 1998) and 7–25 Hz in monkeys (Pellerin and Lamarre, 1997; Courtemanche et al., 2009). However, much less attention has been paid to the suprathreshold (spiking) resonance (Rotstein, 2017). The spiking resonance has been proposed to strengthen input signal processing and data transmission at the theta-frequency band in the GrL (D’Angelo et al., 2001, 2009). In most cases, this feature depends on the spiking mechanisms and the intrinsic properties of single cells (Rotstein, 2017).

Single-neuron responses in the GrL have long been investigated in search of theta-frequency activity patterns (Ros et al., 2009; Gandolfi et al., 2013). Spiking resonance has been claimed to be an intrinsic property of the cerebellar granule cells (GrCs), the most abundant cells not only in the cerebellum but also in the whole mammalian brain (Herculano-Houzel, 2010). Although many experimental studies have registered the electrophysiological activity of single GrCs from rat cerebellar recordings, both from slices *in vitro* (Brickley et al., 2001; Diwakar et al., 2009; Osorio et al., 2010; Delvendahl et al., 2015; Masoli et al., 2017) and *in vivo* (Chadderton et al., 2004; Jörntell and Ekerot, 2006), they have traditionally neglected the presence of spiking resonance. However, only *in vitro* recordings have reported spiking resonance (as enhanced bursting activity) at theta-frequency band of single cerebellar GrCs in response to low-frequency sinusoidal stimulation (D’Angelo et al., 2001; Gandolfi et al., 2013). According to these studies, the spiking resonance could emerge from an intrinsic property of the neurons that selectively enhance low-frequency stimulation responses due to a combination of passive and active membrane properties (Hutcheon and Yarom, 2000; Magistretti et al., 2006; Das and Narayanan, 2017). However, the functional role of resonance at the theta band in the processing of the cerebellar GrCs remains largely unclear.

Computational modeling has demonstrated to be an effective strategy in exploring the origin of resonant behavior in the GrCs. Detailed models (i.e., integrating a high degree of biological plausibility) allowed fine-grained studies about the intrinsic mechanisms involved at isolated GrCs (D’Angelo et al., 2001). Additionally, a conductance-based Hodgkin-and-Huxley (HH) mono-compartmental GrC model evidenced that the subthreshold voltage-dependent potassium current (I_KSlow_) is at the core of the intrinsic resonance during sinusoidal stimulation (Nieus et al., 2006; Solinas et al., 2010; Gandolfi et al., 2013; Rössert et al., 2014; Masoli et al., 2017). However, the high computational cost associated to the simulation of this type of detailed model makes them only suitable for small scale models of the GrL network or short simulations (Nieus et al., 2006; Diwakar et al., 2009; Solinas et al., 2010; Gandolfi et al., 2013).

Thus, simplified models appear to be an exceptional alternative for exploring the functional role of resonant activity in information processing. Simplified models combine computational efficiency and realistic neuronal dynamics. Considering this, the adaptive exponential integrate-and-fire (AdEx) model (Brette and Gerstner, 2005) only includes two coupled differential equations that capture adaptation and resonance properties (Naud et al., 2008), while enabling large scale implementations of neuronal circuits. Although the AdEx model can be seen as a two-dimensional reduction of the spike initiation in HH models, the specific parameter values of the model configuration to match with electrophysiological measurements (Jolivet et al., 2008; Hanuschkin et al., 2010; Barranca et al., 2013; Venkadesh et al., 2018) cannot be experimentally determined as they require an automatic parameter tuning algorithm.

In this article, we present a methodology for the development of simplified neuron models based on the AdEx generic model template that consider both biological relevance and computational efficiency. Evolutionary algorithms (EAs) have been used to find suitable sets of parameters to capture specific firing dynamics. The application to the use case of cerebellar GrC models allows the replication of the most essential properties of the biological cell that are key for the frequency and timing of firing patterns in the neural code. We particularly focus on the spiking resonance of bursts in the theta-frequency band that has been experimentally evidenced in previous studies in the literature. We also address how the inclusion of different spiking properties in the fitness function (FF) affects the behavior of the optimized neuron configuration.

## Materials and Methods

### Neuron Model

The proposed mathematical model of the cerebellar GrC aims to maintain biological realism (to capture important aspects of single-neuron processing) as well as a low computational cost. We have selected the AdEx neuron model (Brette and Gerstner, 2005) as the generic template model. Since GrCs have a compact and simple morphology (D’Angelo et al., 1995, 2001; Delvendahl et al., 2015), a mono-compartment model, such as an AdEx point neuron model, represents a reasonable approach. Previous studies have addressed how this model can be tuned to capture biological realism and compared to more detailed models (Brette and Gerstner, 2005; Nair et al., 2015) as well as recordings in pyramidal neurons, in which this model has been demonstrated to fit, at least qualitatively, a rich set of observed firing patterns (Brette and Gerstner, 2005; Jolivet et al., 2008; Naud et al., 2008).

The AdEx model accounts for only two coupled differential equations and a reset condition regulating two state variables, the membrane potential (*V*) and the adaptation current (*w*), according to the following equations:

(1) $$C_{m}\frac{dV}{dt}=-g_{L}(V-E_{L})+g_{L}\Delta_{T}\exp\left(\frac{V-V_{\text{T}}}{\Delta_{\text{T}}}\right)+I(t)-w$$

(2) $$\tau_{w}\frac{dw}{dt}=a(V-E_{L})-w$$

Equation (1) describes the evolution of the membrane potential (*V*) during the injection of the current [*I(t)*]. When the membrane potential is driven beyond the threshold potential (*V_T_*), then the exponential term of the slope factor (*Δ_T_*) models the action potential (AP). This depolarization ends when the membrane potential reaches the reset threshold potential (*V_peak_*). Then, the membrane potential (*V*) is instantaneously reset to *V_r_* and the adaptation current (*w*) is increased a fixed amount (*b*).

The first term in equation (1) models the passive membrane mechanisms dependent on the total leak conductance (*g_L_*), the leak reversal potential (*E_L_*), and the membrane capacitance (*C_m_*), all regulating the integrative properties of the neuron. The second (exponential) term represents the activation of the sodium channel in a Hodgkin-Huxley type neuron model (Naud et al., 2008), whose dynamics are determined by the parameters *Δ_T_* and *V_T_*. Equation (2) describes the evolution of the recovery variable (*w*). It depends on the adaptation time constant parameter (*τ_w_*) and the subthreshold adaptation (*a*), while (*b*) defines the spike-triggered adaptation. In our simulations, the refractory period (*τ_ref_*) was set to 1 ms. The membrane potential was initially set to the same value as the leak reversal potential (*V_init_* = *E_L_*).

To sum up, 10 parameters define the dynamics of the AdEx neuron model that need to be tuned to reproduce the firing properties of the cerebellar GrCs.

### Model Optimization With Evolutionary Algorithms

Our optimization method is based on an EA that allows multiple parameter exploration to fit the experimentally recorded firing behavior (Jolivet et al., 2008; Hanuschkin et al., 2010; Barranca et al., 2013; Venkadesh et al., 2018). After the execution of the EA, it provides sets of parameters that minimize the FF, i.e., the function which associates each parameter set with a single value quantifying the goodness of such a neuron configuration. Our FF (score) includes a weighted sum of specific features related to spike firing that we consider biologically relevant, according to equation (3).

(3) $$score=\sum_{\text{i}=1}^{n}\left[abs(feat_{i}-\exp_{i})\cdot w_{i}\right]$$

The score is defined as the sum of every firing pattern feature (*i*) in response to the corresponding experimental stimulation protocols. The score of each feature is calculated as the absolute value (*abs*) of the difference between the feature value extracted from the simulated neuron trace with the parameter configuration of the individual (*feat_i_*) and the feature value extracted from the experimental recordings (exp_i_). This is multiplied by the weight associated with each feature (*w_i_*; see “Simulations” section below). The EA will perform progressive parameter optimization to select the individual (set of parameters) that minimizes the fitness value (score). Thus, each individual represents a set of GrC model parameters and the FF quantifies the similarity between the firing pattern in the simulated neuron model and the experimental recording of the neuron in response to the same stimulation protocols.

We also explore an alternative method which aims to evaluate the variability of the burst frequency over successive oscillatory cycles. The score of the burst frequency has been complemented with an additional multiplicative term related to the standard deviation of the burst frequency over consecutive oscillatory cycles, according to equation (4). By using this method as the FF of the burst frequency feature, the EA will prefer neuron model configurations whose burst frequency not only keep close to the target (experimentally recorded) value but are also stable over oscillatory cycles.

(4) $$score_{\text{BF}}=\sum_{j=1}^{N}\left[abs(\bar{BF_{sim_{j}}}-\bar{BF_{\exp_{j}}})\cdot w_{\text{BF}}\cdot (std(BF_{sim_{j}})+1)\right]$$

According to this formula, the score of the burst frequency feature (*score*_BF_) is defined as the sum of each score for all the sinusoidal stimulation frequencies (*N*; 14 sinusoidal frequencies, see Table 3). The individual score for each stimulation frequency is calculated as the absolute difference (*abs*) between the burst frequency (averaged over 10 oscillatory cycles) of the simulated neuron ($\bar{BF_{sim_{j}}}$) and the experimental value at that stimulation frequency ($\bar{BF_{\exp_{j}}}$). This term is multiplied by the weight of the burst frequency feature (*w*_BF_) and the standard deviation of the simulated neuron burst frequency [$std(BF_{sim_{j}})$] plus one.

**Table 1:** Parameters boundaries used for the neuron optimization procedures.

Parameter name (unit)	Fixed boundaries	Parameter name (unit)	Fixed boundaries
*C_m_* (pF)	[0.1, 5.0]	*V_T_* (mV)	[−60, −20]
*Δ_T_* (mV)	[1, 1000]	*a* (nS)	[−1, 1]
*E_L_* (mV)	[−80, −40]	*b* (nA)	[−1, 1]
*V_r_* (mV)	[−80, −40]	*g_L_* (nS)	[0.001, 10.0]
*V_peak_* (ms)	[−20, 20]	*τ_w_* (ms)	[1, 1000]

*The minimum and maximum values of the parameters are indicated in square brackets*.

**Table 2:** Feature and scores obtained with simulated neurons after EA optimization with different fitness functions.

	Burst frequency 6 pA (Hz)					Burst frequency 8 pA (Hz)				
		Simulation						Simulation			
Sinusoidal Stim. Freq. (Hz)	Experimental	*FF1*	*FF2*	*FF3*	*FF4*	Experimental	*FF1*	*FF2*	*FF3*	*FF4*
0.58	41.43	36.77	**37.66**	35.73	35.19	45.00	**45.62**	42.63	45.78	42.68
2.12	49.29	47.36	46.29	**47.52**	46.15	55.71	55.42	**55.75**	56.70	53.97
4.04	54.00	51.78	**52.82**	51.81	50.74	60.00	59.03	61.01	**59.84**	60.39
5.96	59.29	**55.22**	54.32	52.36	53.28	65.71	63.16	**65.57**	63.83	63.07
8.08	55.00	**55.04**	53.93	55.25	54.74	66.43	64.43	**66.23**	64.94	64.52
10.19	45.71	50.51	57.97	**50.48**	55.25	64.29	69.44	68.94	69.93	**67.57**
12.31	-	-	-	-	-	58.57	66.23	**58.62**	66.94	66.01
14.23	-	-	-	-	-	50.00	49.30	71.43	**50.02**	51.74
Score		**20.71**	26.26	**21.61**	28.45		**19.94**	29.90	**19.33**	21.45
	Mean frequency (Hz)					First spike latency (ms)				

		Simulation					Simulation			

Step-current amp. (pA)	Experimental	*FF1*	*FF2*	*FF3*	*FF4*	Experimental	*FF1*	*FF2*	*FF3*	*FF4*
10	30	(1)	**30**	(2)	19	31.90	(45.8)	(9.9)	**36.10**	14.90
16	45	(35)	49	(35)	**45**	19.00	(12.8)	(6.4)	**12.40**	9.00
22	60	(72)	67	(73)	**66**	14.65	(8.5)	(5.0)	**8.40**	6.70
Score		(51)	**11**	(51)	17		(26.25)	(44.25)	**17.05**	34.95

*The score corresponding to different features (burst frequency, IF curve, and latency to the first spike) of the best-performing individuals are shown against the target experimental values. Mean frequency and latency to the first spike experimental values were extracted from Masoli et al. (2017), and experimental recordings of burst frequency from D’Angelo et al. (2001). Note that, although the standard deviation of the burst frequency is considered by the EAs, they are not included in the burst frequency scores to obtain an overall view of the distance between the simulated and experimental values of every feature. The values in brackets correspond to the features not included in the FFs (and are just evaluated for comparison). In bold are the closest values and minimal scores*.

**Table 3:** Parameter values of the best-performing neuron models.

Parameter name (unit)	*FF1*	*FF2*	*FF3*	*FF4*
*C_m_* (pF)	3.10	4.21	3.36	2.80
*Δ_T_* (mV)	5.42	1.09	7.01	22.07
*E_L_* (mV)	−64.06	−51.42	−59.92	−58.00
*V*_peak_ (mV)	−13.49	6.80	−12.24	−17.56
*V_r_* (mV)	−70.28	−73.66	−64.86	−71.31
*V_T_* (mV)	−40.59	−38.00	−40.31	−24.01
*a* (nS)	0.26	0.36	0.36	0.23
*b* (nA)	0.19	0.65	0.15	0.37
*g_L_* (nS)	0.49	0.17	0.67	0.25
*τ_w_* (ms)	327.25	338.75	365.41	619.07

*Parameter values of the individuals resulting from the optimization process with different FFs. Membrane capacitance (*C_m_*), slope factor (*Δ_T_*), leak reversal potential (*E_L_*), reset value for membrane potential after a spike (*V_r_*, map to resting potential *V_init_*), spike detection threshold (*V_peak_*), spike initiation threshold (*V_T_*), subthreshold adaptation (*a*), spike-triggered adaptation (*b*), leak conductance (*g_L_*) and adaptation time constant (τ_w_)*.

For every execution, the EA runs for 50 generations and 1,000 individuals in the population. The initial generation is set with 1,000 simulated neurons with parameters created according to a uniform distribution ranging between the boundaries indicated in Table 1. During each generation of the EA, each model configuration (individual) is simulated and ranked according to the FF (equation 3). The next generation is created using basic genetic operators, such as crossover and mutation. The one-point crossover operator was used with a 60% probability and the uniform mutation operator was used with a 10% probability. In those individuals randomly selected to mutate, each parameter was mutated with a probability of 15%. To select those individuals to be included in the population in the next generation, the selection was carried out by three-individual tournaments. Therefore, the new population is composed of the winners (minimum score) resulting from 1,000 tournaments with randomly-chosen individuals. Finally, the individual with the minimum score obtained during each complete EA execution is selected as the best neuron model (the most suitable parameter configuration to the target behavior).

### Fitness Functions and Feature Quantification

#### Biological Data Used as Reference

**Figure 1:**
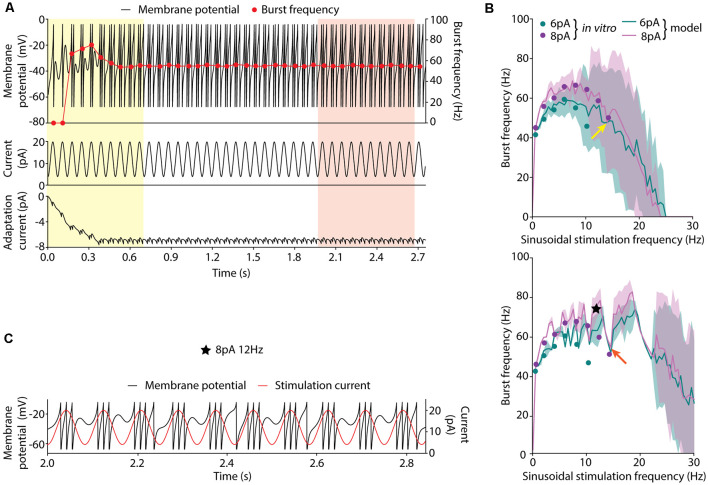
Burst frequency analysis. **(A)** Membrane potential (*V*; top, black line, left axis), burst frequencies (top, red line, right axis), stimulation current (middle) and adaptation current (*w*; bottom) of a neuron model instance stimulated with the sinusoidal current of 8-pA amplitude, 12-pA offset current and 14.23-Hz frequency. Two strategies for measuring the average burst frequency are shown: (i) during the initial 10 cycles (yellow shadow); and (ii) 10 cycles after 2 s of initial stabilization (red shadow). **(B)** Burst frequency in response to sinusoidal stimulation with different stimulation frequencies (in steps of 0.5 Hz) up to 30 Hz, 12-pA offset current and 6-pA (green), or 8-pA (purple) amplitude measured during the initial cycles (top) and after 2-s stabilization period (bottom). The solid lines represent the average burst frequency from the simulated neuron model configuration and the shaded area shows the standard deviation. The arrows indicate the average burst frequency corresponding to the stimulation frequency shown in **(A)** during the initial cycles (yellow arrow, 49.74 ± 25.81 Hz) and after 2 s of stabilization (red arrow, 55.04 ± 0.38 Hz). The dots correspond to the burst frequency data obtained from *in vitro* recordings of cerebellar GrCs in D’Angelo et al. (2001) with the same stimulation protocols (Table 2). The black star indicates the point further explored in plot C. **(C)** Simulated membrane potential (*V*; black line, left axis) during 10 oscillatory cycles selected after 2-s initial stabilization in response to 12-Hz sinusoidal stimulation. The red line (right axis) represents the stimulation current signal (8-pA amplitude and 12-pA offset).

**Figure 2:**
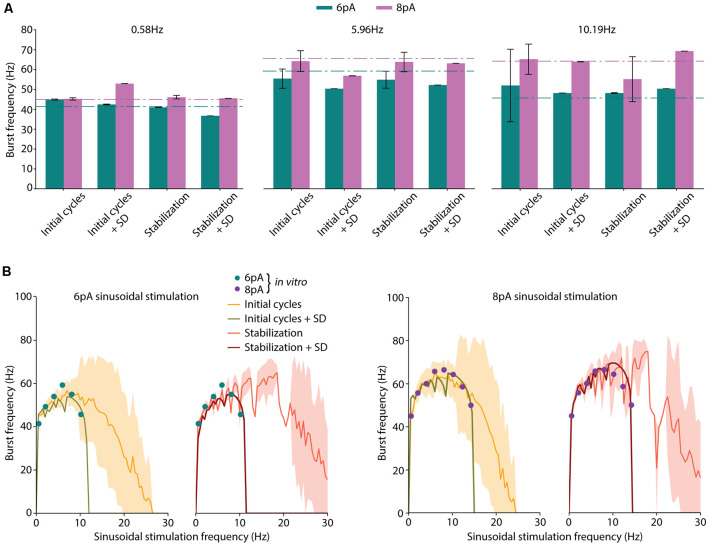
Neuron model optimization with different strategies. **(A)** Average burst frequencies in response to low (0.58 Hz), medium (5.96 Hz), or high (10.19 Hz) sinusoidal stimulation frequencies. The mean value over 10 oscillatory cycles and the standard deviation from the best performing individual are represented for each considered fitness function (FF). Corresponding *in vitro* values from D’Angelo et al. (2001) for each stimulation frequency and amplitude (6 pA, green and 8 pA, purple) are shown as dashed-dotted lines. **(B)** Spiking resonance curves in response to 6 pA and 8 pA of sinusoidal stimulation. The stimulation frequency of the individuals ranges from 0.5 Hz to 30 Hz with 0.5-Hz steps. Standard deviation is shown shaded in yellow for individuals optimized with *initial cycles* FF, and in red for individuals optimized with the *stabilization* FF. Those individuals optimized including the standard deviation in their score (namely, *initial cycles + SD* and *stabilization + SD*) obtained near-zero standard deviation (this is indeed represented in the green and dark red shaded areas but are hardly visible). The available *in vitro* data extracted from D’Angelo et al. (2001) and specified in Table 3 are shown with colored dots.

The experimental data used as a reference for EA optimization are taken from two different sources (D’Angelo et al., 2001; Masoli et al., 2017). In particular, the burst frequency in response to sinusoidal current stimulation is obtained from D’Angelo et al. (2001). The authors recorded cerebellar GrCs in acute cerebellar slices obtained from 20 ± 2-day-old rats. The slice preparation and whole-cell patch-clamp were performed as reported previously (see their references). The presence of bicuculline prevented GrC rhythmic inhibition by Golgi cells and that spontaneous EPSPs were too rare to affect spike generation. Injection of sinusoidal currents at various frequencies (0.5–40 Hz) revealed resonance in burst spike frequency in correspondence with the positive phase of the stimulus. Spike frequency within bursts increased and then decreased according to the injected current frequency showing spiking resonance. The preferred frequency was 6 Hz with sinusoidal currents of 6-pA amplitude, and 8 Hz with 8-pA amplitude (reference dots in Figures 1B, 2B, 4A, 6). It is important to highlight that in these *in vitro* recordings, the burst frequencies with stimulation frequencies beyond 10.19 Hz in 6-pA amplitude and 14.23 Hz in 8-pA amplitude fell to zero as one or no spikes were obtained.

**Figure 3:**
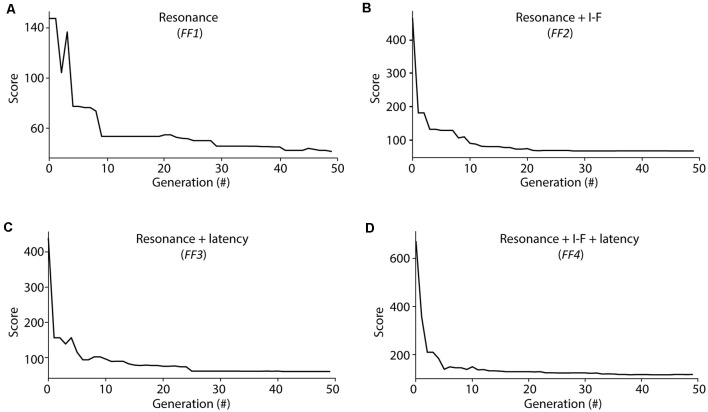
Score evolution during Evolutionary algorithm (EA) optimization. Evolution of the minimal score from the individuals considered at each generation during the optimization processes of EAs configured with different fitness functions (FFs; as described in Methods section): **(A)** *FF1* (Resonance), **(B)** *FF2* (Resonance + I-F curves), **(C)** *FF3* (Resonance + Latency to the first spike), **(D)** *FF4* (Resonance + I-F curves + Latency to the first spike). The score was calculated as the weighted sum of the individual feature scores considered for each FF (equations 3 and 4).

**Figure 4:**
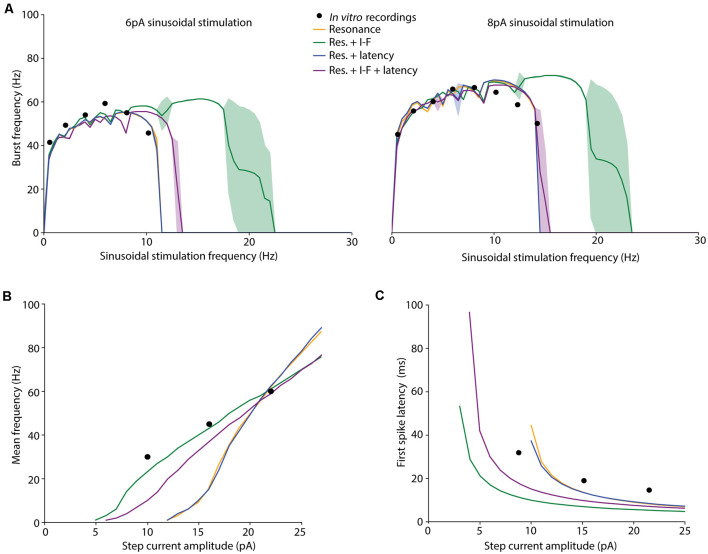
Spiking dynamics of the individuals resulting from different EA implementations. Simulated features of the selected (best fitted) neuron models obtained from the EAs configured with different FFs: only burst frequency (yellow), burst frequency and intensity-frequency (I-F) curve (green), burst frequency and first-spike latency (blue), and burst frequency, I-F curve and first-spike latency (purple). The target *in vitro* data from D’Angelo et al. (2001) and Masoli et al. (2017); specified in Table 3) are represented as black dots. **(A)** Burst frequency in response to 6-pA (left) or 8-pA (right) sinusoidal stimulation with varying frequencies (in steps of 0.5 Hz). The lines represent the simulated average burst frequency over 10 cycles after 2 s of initial stabilization, and the standard deviations are shown as shaded areas. **(B)** I-F curves of the neuron models obtained from the EAs configured with different FFs. The Y-axis represents the average firing frequency in response to 1 s-step-currents. **(C)** Latency to the first spike in response to 1-s-step-currents.

**Figure 5:**
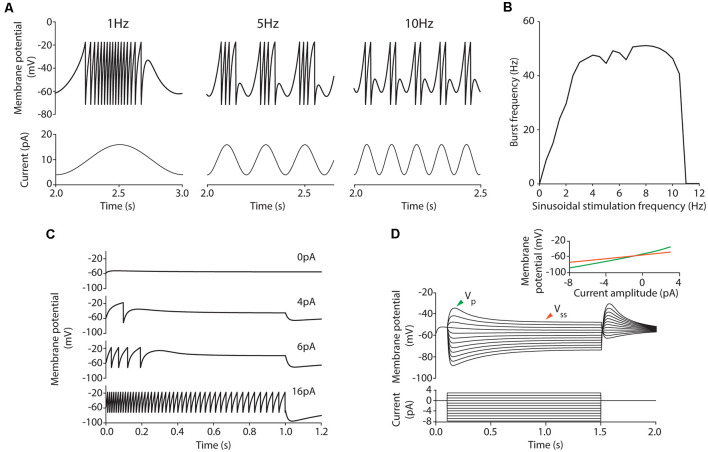
Intrinsic properties predicted by the cerebellar granule cell (GrC) model. **(A)** Neuron model simulation in response to sinusoidal current injection of 10-pA offset and 6-pA amplitude. Bursts are shown after 2 s of stimulation (stabilization). **(B)** Resonance curve (showing burst frequency) in response to the same stimulation protocol. **(C)** Membrane potential evolution in response to 1-s step-current injections with variable amplitude. **(D)** Step pulse hyperpolarizing and depolarizing currents in the subthreshold regimen (from −8 pA to 3 pA in steps of 1 pA from 100 ms to 1500 ms) cause the membrane potential to reach an early peak followed by a decayed (sag response) at a stable level. Current-voltage relationship plots (I–V plots) from the voltage peak (sag; green colored) and from the steady-state (considered at 1,000 ms; orange-colored) demonstrate the absence of inward rectification in the model during the polarization with both hyperpolarizing and depolarizing currents.

**Figure 6:**
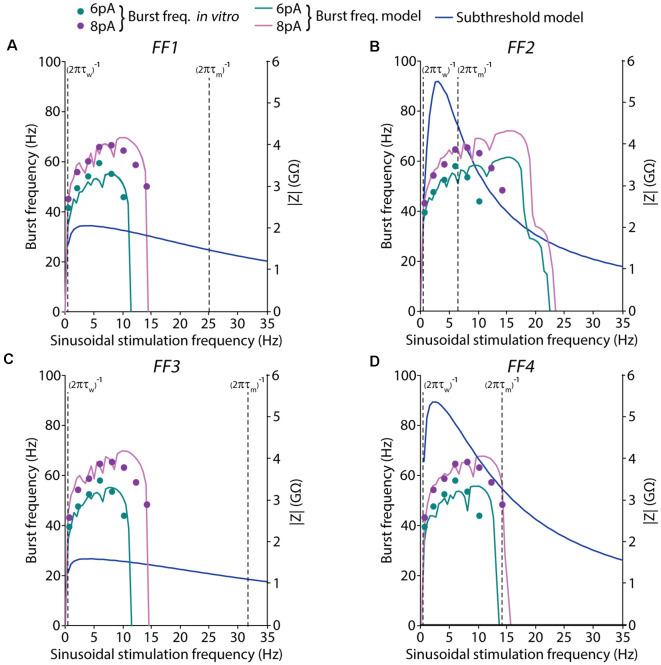
Spiking and subthreshold resonance properties obtained with the models resulting from different EA implementations. Simulated resonance properties under suprathreshold and subthreshold regimes of the selected (best fitted) neuron models obtained from the EAs configured with different FFs: **(A)** only burst frequency, **(B)** burst frequency and I-F curve, **(C)** burst frequency and first-spike latency, and **(D)** burst frequency, I-F curve, and first-spike latency. Burst frequencies are shown in response to 6-pA (green) or 8-pA (pink) sinusoidal stimulation with varying frequencies (in steps of 0.5 Hz). The pink and green lines represent the simulated average burst frequency over 10 cycles after 2 s of initial stabilization (left axis). The target *in vitro* data, from D’Angelo et al. (2001), of the burst frequencies, are represented as dots. Subthreshold resonance properties are represented as the module of the impedances that were calculated with the FFT algorithm in response to 2-pA (blue lines) sinusoidal current stimulation with varying frequencies (in steps of 0.5 Hz; right axis). The cut-off frequency of the high-pass filter is represented as a vertical dashed line in the form of 1/(2*πτ*_w_), while the cut-off frequency corresponding to the low-pass filter is represented as a vertical dashed line in the form of 1/(2*πτ*_m_).

On the other hand, the average firing rate and first-spike latency in response to current pulses were obtained from Masoli et al. (2017). In this case, the authors performed *in vitro* patch-clamp recordings under step current injections. They recorded cerebellar GrCs in acute cerebellar slices from 21-day-old rats.

#### EA Fitness Functions (FFs)

The FF described in equation (3) weights the similarity of different quantified features with the experimental recordings (i.e., the value of the feature extracted from the traces). Since our models aim to reproduce the spiking resonance of bursting, it is required to estimate this resonance as a set of values. Thus, the burst frequency under sinusoidal current injection was calculated as the inverse of the average inter-stimulus interval (ISI) of the output neuron (the cerebellar GrC) during each stimulation cycle. Then, the average burst frequency was measured throughout 10 consecutive cycles of sinusoidal stimulation. The total simulation time was set to 22.5 s. Sinusoidal amplitude values of 6 pA and 8 pA (in addition to 12-pA offset) were used according to the available experimental data (specified in the subsection above), generating spike bursts in correspondence with the positive phase of the stimulus (sinusoidal phase of 270°). To reproduce the differential effect of the oscillatory stimulation frequency, the burst frequency in response to its stimulation frequency was included in the EA as an individual feature (all of them equally weighted). As it occurred in the *in vitro* recordings [see “Biological Data Used as Reference” section above], we have set the burst frequency to zero when the same firing pattern (one or no spike per cycle) has been obtained in the simulated neurons.

Although the firing dynamics of cerebellar GrCs are complex, these cells implement a mechanism of linear frequency encoding through repetitive firing discharge under current stimulation which might help to sustain the spiking resonance of burst frequency at the theta-frequency band (D’Angelo et al., 2001, 2009). Recent literature has characterized the fast repetitive discharge in the GrCs based on the mean frequency (the number of spikes divided by the stimulation time) and the latency to first spike (time of the first spike firing) in response to three different step-current injections (10 pA, 16 pA, and 22 pA) of 1-s stimulation (Masoli et al., 2017).

The optimizations were carried out with FFs that considered different combinations of the minimal number of features that characterize the typical firing of cerebellar GrCs: (1) burst frequency in response to different sinusoidal current stimulations (stimulation at different frequencies of the sinusoidal current); (2) burst frequency feature (as in point 1) in addition to the average mean frequency in response to step-currents; (3) the burst frequency (as in point 1) and the latency to the first spike under step-current stimulations; and (4) all the previous features (burst frequency under sinusoidal stimulation, mean frequency and latency to the first spike under current stimulations; Table 3). Later on, we will refer to these combinations of features as FFs from 1 to 4.

### Simulations

The score of each individual approaches zero as the measured firing features approximate target values. We ran each optimization protocol (EA algorithm) with five different seeds and selected the individual with a minimal score from those executions. The weight of the burst frequency and the mean frequency features were set to 1 (as they both were measured in Hz and present values in comparable scales). The latency to the first spike feature was weighted to 1,000 as it was measured in seconds. Thus, our algorithm equally weights 1 Hz-error in the average mean frequency feature and 1 ms-lag in the latency to the first spike feature.

The EA algorithm was implemented using the DEAP library (Fortin et al., 2012) for Python (version 2.7.12). The GrC model was simulated using NEST (version 2.14.0; Peyser et al., 2017). The model uses the embedded 4th order Runge-Kutta-Fehlberg solver with adaptive step-size to integrate the differential equations. The simulations were run in parallel with SCOOP on a 6-cores 3.30 GHz CPU (32 GB RAM) PC allowing each optimization protocol to run (five simulations with different seeds) in around 7 h.

## Results

### Bursting Frequency Optimization

We conducted preliminary experimentation to determine the best strategy to measure the burst frequency in response to sinusoidal stimulation. As a first approach, we calculated the average burst frequency during the 10 initial cycles of the simulation (as explained in the Methods section). The EA was set to minimize only the error of the average burst frequency in response to all the available data. The resulting neuron model configuration (individual) showed high instability (i.e., highly variable burst frequency) during the initial cycles (Figure 1A, yellow shaded area in the top plot). The same optimization was carried out with five different random seeds and all the individual winners showed similar behavior. Particularly, it can be observed that in response to high stimulation frequencies (namely, 10–14 Hz), the burst frequency remained unsteady for eight oscillatory cycles (Figure 1A, red dots in the top plot). This observation can be explained based on: (i) the model configuration emerging from the EA combines high membrane capacity (*C_m_* ranging between 2.63 and 4.87 pF), low leakage conductance (*g_L_* ranging between 0.49 and 4.31 nS) and low initial membrane voltage (*V*_init_ equal to *E*_L_ as specified in the “Materials and Methods” section; both ranging between −58.18 and −49.88 mV), so that the membrane potential required between cycles 1 and 4 until stable values were reached for several consecutive cycles (Figure 1A, yellow shaded area in the top plot); and (ii) the neuron configuration included long adaptation time constants (*τ_w_*) so that the adaptation current (*w*) required six cycles to reach steady-state (Figure 1A, yellow shaded area in the bottom plot). Although the average burst frequency stays close to the experimental measures for every stimulation frequency, the standard deviation of the burst frequency over the measuring cycles is higher than desired, especially for high stimulation frequencies (Figure 1B, top plot).

Aiming to overcome the instability produced during the initial period of simulation, we tested whether averaging over 10 bursts (oscillatory cycles) after 2 s of initial stabilization produced different results. This period was chosen as it corresponds to twice the maximum allowed adaptation time constant (*τ_w_*; see Table 1). In this way, the neuron membrane potential reached steady state (Figure 1A, red shaded area at top plot) before measuring and averaging the burst frequency. Not unexpectedly, the EA set with this second estimation method resulted in neuron configurations whose average burst frequencies closely matched the experimental measures (Figure 1B, green and purple lines in the bottom plot). However, the standard deviation of the burst frequencies remained higher than desired (although remarkably lower than using the first estimation method) in response to high stimulation frequencies (Figure 1B, green, and purple shaded areas in the bottom plot). E.g., when stimulated with 14.23 Hz (Figure 1B, red arrow in the bottom plot) the average burst frequency is stable with almost no standard deviation (55.04 ± 0.38 Hz; Figure 1A). On the contrary, when stimulated with 12 Hz (Figure 1B, a black star at bottom plot) the simulated neuron showed an increased standard deviation of the average burst frequency (74.96 ± 5.99 Hz). It occurred because the neuron did not fully recover from one oscillatory cycle to the next one (Figure 1C). This situation produces enhanced variability in the burst frequency values for some neuron configurations. To prevent this issue, we set the EA with a third method for calculating the score of each individual based on the 2-s-stabilization method (described in the “Materials and Methods” section).

Four implementations of FFs were considered once defined the period considered for burst frequency calculation and the inclusion of penalization for instability: (1) average burst frequency calculation over initial cycles (shortly, *initial cycles*); (2) average burst frequency calculation over initial cycles with the penalization of the standard deviation (shortly, *initial cycles + SD*); (3) average burst frequency calculation after 2-s simulation (shortly, *stabilization*); and (4) average burst frequency calculation after 2-s stabilization period with the penalization of the standard deviation (shortly, *stabilization + SD*). The score was calculated according to equation (3) in the cases of *initial cycles* and *stabilization* (cases 1 and 3), and according to equation (4) in the cases of *initial cycles + SD* and *stabilization + SD* (cases 2 and 4).

The EA was executed five times for each case considering different random seeds to obtain the neuron model configurations that best matched the experimental values of average burst frequencies (Table 3). The individuals with the lowest score were selected. Not unexpectedly, all the individuals presented similar values to the experimental data (Figure 2A), validating the operation of the EA. In response to low (0.58 Hz) stimulation frequency, negligible standard deviations were obtained with all the considered FFs (left plot in Figure 2A). However, higher stimulation frequencies (i.e., 5.96 Hz and 10.19 Hz) resulted in increased standard deviations for those functions which did not include SD penalization (namely, *initial cycles* and *stabilization*; Figure 2A, middle and right plots, respectively).

We then compared the spiking resonance curves of the individuals obtained using each FF (Figure 2B). When the penalization of standard deviation is not included in the FF (namely, *initial cycles* and *stabilization*), the average burst frequencies (Figure 2B, yellow and orange lines) are near the experimental values (the sum of the distances between simulated and experimental burst frequency features are 19.44 Hz and 43.15 Hz in *initial cycles* and *stabilization*, respectively; Figure 2B, colored dots), but with large standard deviation (the sum of the SDs of burst frequency features are 104.62 Hz and 43.44 Hz in *initial cycles* and *stabilization*, respectively; Figure 2B, yellow and orange shadow). Additionally, resonance curves fall to zero (indicating one or zero spikes per oscillatory cycle) with remarkably higher stimulation frequencies (beyond 25 Hz), especially with the *stabilization* function. Thus, the model configuration resulting from the usage of the *initial cycles* FF appropriately reproduced spiking resonance at theta-frequency band but with considerable variability. Differently, the *stabilization* FF drove to the neuron models whose resonance peaks were beyond the theta-frequency band (around 20 Hz). This situation makes these two neuron models unsuitable for our aim.

When the penalization of standard deviation is included in the FF (namely, *initial cycles + SD* and *stabilization + SD*), average burst frequencies are also close to the experimental data (the sum of the distances between simulated and experimental burst frequency features are 50.79 Hz and 41.50 Hz in *initial cycles + SD* and *stabilization + SD*, respectively; Figure 2B, green and dark red lines) and they are stable, with almost negligible standard deviations (the sum of the SDs of burst frequency features are 0.69 Hz and 1.05 Hz in *initial cycles + SD* and *stabilization + SD*, respectively; Figure 2B, green and dark red shadow areas representing the standard deviations that are almost negligible and hardly visible in the plots). Interestingly, the neuron models resulting from these individuals show resonance curves falling to zero just above the last stimulation frequency points (10.19 Hz at 6-pA and 14.23 Hz at 8-pA sinusoidal stimulations) as was experimentally tested in real GrCs, generating one or no spikes per cycle at higher frequency sinusoidal stimulations (Figure 2B, green and dark red lines; D’Angelo et al., 2001). Thus, these model configurations are considered to better reproduce the spiking resonance at the theta-frequency band.

According to these preliminary results, it is preferable to calculate the burst frequency after the 2-s-stabilization period and including the standard deviation as part of the FF (*stabilization + SD*; named *FF1* in the next subsection). This FF drives our EA to penalize unstable configurations, resulting in neuron model configurations that match the spiking resonance at the theta-frequency band of biological cerebellar GrCs and maintain stable neuronal behavior during the oscillatory cycles.

### Parameter Fitting With Other Suprathreshold Dynamics

Once, we had explored the most convenient definition of FF for burst frequency feature optimization, we aimed to demonstrate whether additional electrophysiological properties could also be optimized and reproduced by the neuron model. Thus, we considered other representative firing properties of GrCs that are seemingly relevant in neural transmissions such as the intensity-frequency (I-F) curve and the latency to the first spike in response to different stimulation currents. We carried out additional optimization experiments with different combinations of features in the FF: burst frequency under sinusoidal stimulation (namely, *FF1*), burst frequency under sinusoidal stimulation and mean frequency (I-F) under step current injection (namely, *FF2*), burst frequency under sinusoidal stimulation and latency to the first spike in response to step current injection (namely, *FF3*), and all the three mentioned features together (namely, *FF4*).

The evolution of the minimum score of the individuals in the explored population from these EAs showed fast convergence during the optimization processes among generations (Figure 3). We aim to determine whether the usage of different FFs affect the capability of the resulting neuron models to resonate in the theta-frequency band as well as determining whether the proposed AdEx model can reproduce all these different firing features in a single parameter configuration. The scores obtained from the evaluation of all the features (included or not in its EA implementation) simulated by the individuals are shown in Table 2, and the corresponding parameters of the best performing individuals with each EA configuration are in Table 3.

#### Spiking Resonance in the Theta-Frequency Band

Cerebellar GrCs have been demonstrated to resonate in a rather broad theta-frequency band. The spiking resonance peak has been described around 6–12 Hz (D’Angelo et al., 2001) in experimental measurements, and around 4–10 Hz in previous detailed GrC models (D’Angelo et al., 2001, 2009; Magistretti et al., 2006; Gandolfi et al., 2013; Masoli et al., 2017). The proposed EAs selected neuron models matching the burst frequency of the experimental curves when the configured FFs included only the burst frequency (*FF1*; preferred resonance frequency within 7–11 Hz), the burst frequency and the latency to the first spike (*FF3*; preferred resonance frequency within 7–11 Hz) and all the three features considered in this work (*FF4*; preferred resonance frequency within 8–12 Hz; Figure 4A). The simulation of the selected individuals closely fitted the experimental data with stable burst frequencies (between 0.5–1.5 Hz SD) and burst frequency falling to zero (one or zero spikes per cycle) with stimulation frequencies beyond 10.19 Hz (6-pA amplitude) and 14.23 Hz (8-pA amplitude), respectively.

The best-fitted individuals for the experimental spiking resonance were the neuron model resulting from the EA with *FF1* (the sum of the distances between simulated and experimental burst frequency features is 40.65 Hz) and the neuron model from the EA with *FF3* (the sum of the distances between simulated and experimental burst frequency features is 40.94 Hz), closely followed by the neuron model from the EA with *FF4* (the sum of the distances between simulated and experimental burst frequency features is 49.90 Hz) and, finally, the neuron model from the EA with *FF2* (the sum of the distances between simulated and experimental burst frequency features is 56.16 Hz). Not unexpectedly, the EAs with FFs which included only burst frequency features resulted in neuron models with the best fitting of the resonance to the experimental data. On the contrary, the individuals resulting from EAs with the FF that included all the features (*FF4*) showed a shifted resonance curve only with 6-pA-amplitude sinusoidal stimulation (Table 2 and Figure 4A, purple line). The individuals resulting from the EAs with the FF including burst frequency and I-F curve (but not first spike latency; *FF2*) showed resonance beyond the theta range (Figure 4A, green-shaded lines) with unstable behavior (large standard deviations).

#### I-F Curve

We also evaluated the membrane voltage response while injecting step currents of increasing amplitude. Beyond specific thresholds of injected current fast repetitive firing was reproduced (Figure 4B). The individual resulting with *FF1* and *FF3* showed rheobases (understood as the minimum current injected required to fire a single AP) at 10 pA, the individual resulting with *FF2* at 3 pA, and the individual resulting with *FF4* at 4 pA. This is in agreement with the experimental rheobases obtained for GrCs (ranging between 2 pA and 10 pA; D’Angelo et al., 2001; Bezzi et al., 2004; Gandolfi et al., 2013; Masoli et al., 2017). The best fitting to the experimental frequency values were obtained, as expected, by the neuron models resulted from those FFs that included the I-F curve in their features to optimize (*FF2* and *FF4*; Table 2 and in Figure 4B, green and purple lines).

Linear coding of stimulus intensity (I-F curve) is usually used as a measure of the intrinsic excitability of GrCs. I-F plots were constructed (using 1-s current stimulation with amplitude ranging between the rheobase and 25 pA; Figure 4B) and fitted to a linear function (*r* > 0.9). The slope of such a linear function is usually representative of the intrinsic excitability of the neurons. The output frequency values of the neuron model with the FF containing all the features (*FF4*) were slightly further away from the experimental values (slightly higher score; see mean frequency in Table 2) than those resulting from the EA with the FF including burst frequency and mean frequency (*FF2*). However, their I-F slopes were very similar (3.83 Hz/pA and 3.39 Hz/pA, respectively) and near the slope of the experimental points used in the EAs (2.5 Hz/pA, which was calculated from the experimental frequency values in Table 2). The neuron models that were not optimized for this frequency (using *FF1* and *FF3*) resulted in higher scores (their firing frequencies fell far from the experimental points) and higher I-F slopes (6.27 Hz/pA and 6.36 Hz/pA, respectively; Figure 4B, yellow and blue lines). Despite this, their I-F slopes were coherent to those slopes reported in previous GrC models (7 Hz/pA in D’Angelo et al., 2001; Bezzi et al., 2004; Masoli et al., 2017). Both the I-F slope ranges obtained (around 3.5 Hz/pA and around 6.3 Hz/pA) are then considered biologically plausible since they fall within the experimentally recorded values (6.5 ± 3.2 Hz/pA in D’Angelo et al., 1995).

#### Latency to the First Spike

Another central behavior of biological GrCs is that the latency to the first spike decreases and spike frequency increases when the injected current intensity is increased (D’Angelo et al., 2001). Similar behavior is observed in the neuron models resulting when using all the proposed FFs (Figure 4C). Experimental *in vitro* recordings evidenced that the latency to the first spike decreased from 31.9 ± 16.2 ms with 10-pA step current to 14.65 ± 9.4 ms with 22-pA current (Masoli et al., 2017). The closest latencies to these data were obtained by the neuron model resulted from the EA with the FF that included first-spike latency in its definition (*FF3*; Figure 4C, blue line). The neuron model resulting from the EA with *FF1* (Table 2 and Figure 4C, yellow line) obtained fitted results too. Differently, those individuals from the EA with FFs that included the step-current firing rate (*FF2* and *FF4*) generated higher latencies than those reported, mainly with low stimulation currents (Figure 4C, green and purple lines). The individual from the EA with *FF2* reproduced the closest fitting to the I-F curve but the least suitable to fit either the theta-frequency band or latency to the first spike. However, according to other *in vitro* recordings (D’Angelo et al., 1995, 1998; Brickley et al., 1996; Cathala et al., 2003) and computational GrC models (D’Angelo et al., 2001; Diwakar et al., 2009; Masoli et al., 2017), latencies to the first spike decreased from around 100 ms at the rheobase to around 1 ms, similar to that from the individual resulting from the EA with the *FF4* (see first spike latency in Table 2).

### Selecting a Biologically Plausible GrC Model

Overall, the most accurate neuron model according to all the features (with the lowest sum of the distances between experimental and simulated features of burst frequency, mean frequency and first spike latency) corresponded to the individual obtained including all the features in the EA (the sum of the distances is 101.85 using *FF4*), followed by the neuron model obtained from the inclusion of the average burst frequency and latency to the first spike (the sum of the distances is 108.99 using *FF3*). The individual resulting from the FF only defined by the average burst frequency had, unexpectedly, a higher total score (the sum of the distances is 117.9 using *FF1*) than the individual from the FF of average burst frequency and I-F curve (the sum of the distances is 111.41 using *FF2*). Therefore, the simplified model configuration with the best fitting to the spiking dynamics of a real GrC is the individual resulted from the EA implementation that contains all the spiking properties (namely, *FF4*).

The behavior of this model is presented in Figure 5. When stimulated by just-threshold sinusoidal stimulation, the model generated spikes clustered in doublets-triplets or longer bursts (as in D’Angelo et al., 1998, 2001; Gandolfi et al., 2013; Figure 5A) with specific tuning in the theta frequency band (7–10 Hz; Figure 5B). In response to step-current stimulations, the model resulted in regular spike discharge (Figure 5C) with latency compatible with the experimental data in real cells. Additionally, the model exhibited other emergent properties (i.e., not selected during the EA optimization). First, the neuron is silent at rest (Figure 5C). When stimulated by depolarizing step-current injections, the neuron model elicited a single spike with 4 pA as in D’Angelo et al. (2001). The firing rate showed no adaptation with 0, 4, and 6 pA and little adaptation with 16 pA which is similar to the experimental recordings (Masoli et al., 2017; Figure 5C). However, we evaluated some other emergent properties from the subthreshold regime typical of a cerebellar GrC, such as the inward rectification (D’Angelo et al., 1995). The model did not reproduce the inward rectification during the application of current steps in the hyperpolarizing direction neither its I-V relationships (Figure 5D). Simulations using detailed neuron models based on *in vitro* recordings suggested that some well-demonstrated features of the intrinsic excitability of cerebellar GrCs—namely fast repetitive firing, oscillations, bursting and resonance in theta-range—had in common the dependence upon the same mechanism (a slow K^+^ current component; D’Angelo et al., 2001; Gandolfi et al., 2013). However, the inward rectification of a cerebellar GrC was fully explained by another type of current (a fast K-dependent inward rectifier; D’Angelo et al., 2001). Despite there is evidence that an exponential integrate and fire model can fit and reproduce deflective I-V curves in the near-threshold range (Badel et al., 2008), it seems complicated to obtain an AdEx model configuration able to fully reproduce all these different behaviors in different regimes (suprathreshold and subthreshold, respectively) with a single set of parameters configuration (and especially considering that the optimization algorithm only fitted the spiking dynamics).

### Bursting Resonance vs. Subthreshold Resonance in AdEx Neuron Models

The formal analysis of the resonance in integrate-and-fire neuron models represents a well-studied field. However, how this resonance extends to the suprathreshold regime is still under exploration. The subthreshold intrinsic resonance in a biological neuron is shaped by the dynamics of voltage-gated ionic currents, which can be expressed in variable levels but may have a stable resonant frequency (Fox et al., 2017). The resonant frequencies result from a combination of low-pass and high-pass filter mechanisms produced by the interplay of the passive membrane properties and one or more ionic currents and their interaction with the oscillatory inputs (Hutcheon and Yarom, 2000; Fox et al., 2017). The slow resonant currents (or currents having resonant gating variables) oppose voltage changes and act as high-pass filters. Finally, fast amplifying currents (or currents having amplifying gating variables) favor voltage changes and can make resonance more pronounced (Hutcheon and Yarom, 2000; Fox et al., 2017).

One of the main advantages of the AdEx model is the low computational requirements derived from accounting only two differential equations (and state variables; equations 1 and 2). The AdEx model describes a capacitive current (*CdV/dt*) balanced by membrane currents compressed in three terms: (1) the leak current describes the passive membrane properties and determines an equivalent low-pass filter according to the membrane time constant (Hutcheon and Yarom, 2000); (2) the exponential term describes the activation-dependent on the Na^+^ voltage; and (3) the adaptation current, which has proven effective in reproducing more complex subthreshold dynamics such as resonance (Richardson et al., 2003; Brette and Gerstner, 2005; Badel et al., 2008; Naud et al., 2008). The adaptation current could implement a high-pass filter (representative of slow voltage-gated current). This high-pass filter needs to have slow activation according to the adaptation time constant (*τ_w_*), which drives it to turn on or off with a relative delay with respect to the passive membrane charge.

Many types of neurons show membrane potential resonance through a peak in the impedance in contrast with the frequency curve (Z-profile; Fox et al., 2017). The resonance frequency peak can be estimated depending on the adaptation time constant from the high-pass filter [cut-off frequency defined as 1/(2*πτ*_w_)] and the membrane time constant from the low-pass filter [cut-off frequency defined as 1/(2*πτ*_m_), where *τ*_m_ = *C*_m_/*g*_L_].

To better understand the oscillatory behavior of our resulting AdEx models, we have explored how resonance frequencies relate in both the subthreshold and suprathreshold regimes. To analyze the subthreshold resonance, we used the impedance profile measured as the amplitude of the membrane voltage response to sinusoidal current stimulation with different frequencies (applying the Fast Fourier Transform algorithm). We have compared both (subthreshold and suprathreshold) resonant peaks and evaluate if both falls within the cut-off frequencies range of the high-pass and low-pass filters that control subthreshold resonance.

In all the models under study, the resonance peaks resulting from the subthreshold regime fall remarkably far from the preferred frequency in the suprathreshold regime. In the case of individuals optimized using *FF1* and *FF3* (Figures 6A,C), spiking resonance peaks around theta-band (10 Hz) while subthreshold resonance peaks are under 3 Hz. Both types of resonance fall into the wide window between their low-pass and high-pass filters. Even further, the neuron models obtained from the optimizations using *FF2* and *FF4* showed subthreshold resonance profiles notably sharper. However, the neuron model *FF2* showed a spiking resonance peak markedly shifted to higher frequencies (as it was highlighted in Figure 4A and addressed in the “Discussion” section), which falls out of the band between low-pass and high-pass cut-off frequencies (Figure 6B). On the other hand, the neuron model *FF4* showed spiking resonance peak around theta-band that falls into the interval between the low-pass and high-filter cut-off frequencies.

## Discussion

Computational models represent an essential strategy in neuroscience for researching the function of certain neuronal properties which remain insufficiently explored, as is the case of cerebellar resonance in the theta frequency band (4–12 Hz; Buzsáki, 2006). The convenience of having single-compartment GrC models (point neuron models) reconstructing this behavior with both biological realism and computational efficiency represents an initial step towards understanding these firing dynamics and their involvement in the cerebellar synchronization and learning. This study develops a methodological workflow and explores the best alternatives (in terms of FFs and biological features defined in them) for creating simplified models through optimization of their parameters using EAs. As a result, a set of efficient cerebellar GrC models that closely reflect realistic spiking dynamics are proposed.

The suggested methodology has shown to be successful in generating efficient neuron models capturing the fundamental properties of firing in real cells (e.g., cerebellar GrCs). Interestingly, just the inclusion of the burst frequency as an optimization criterion resulted in neuron models essentially reproducing the main properties of a biological GrC neuron. This seems to suggest that this property is dependent on the main parameters of the cell model and can thus be considered a pivotal property that integrates the main features of the GrC. In addition to this, the optimized neuron models proved suitable against a set of properties that could be relevant in neural information transmission and can be used as features for neuron model optimization. They include linear frequency coding (implemented as repetitive firing under step-current stimulation; D’Angelo et al., 2001, 2009) and the latency to the first spike upon current injection (D’Angelo et al., 1995, 2001; Masoli et al., 2017).

The resulting simplified model evidenced electrical properties characteristic in a biological GrC that were not explicitly integrated into the FF. These are rare spontaneous activity (Chadderton et al., 2004; Jörntell and Ekerot, 2006; Rössert et al., 2014) with high attainable spike frequency (low current needed for the spike generation; D’Angelo et al., 1995, 2001) and non-adapting spike discharge with high firing frequencies (D’Angelo et al., 1995, 1998; Brickley et al., 1996; Chadderton et al., 2004). The proposed model also reproduced properties not so closely related to the firing pattern, such as a strong inward rectification [as in the detailed models of D’Angelo et al., 2001; Masoli et al., 2017, *in vivo* (Chadderton et al., 2004) and *in vitro* (D’Angelo et al., 1995) experiments]. These emergent properties were predicted uniquely from the suitability of the whole set of AdEx parameter values. This reinforces the biophysical plausibility (in terms of realistic firing dynamics) of the model with very low computational costs. These results make this neuron model of cerebellar GrC a good candidate for large-scale simulations of realistic networks and analysis of these spiking properties.

According to our results, a single set of parameters (specific configuration) of the AdEx model can reproduce a variety of spiking features (wrapped in the FF), but also some emergent behaviors (not explicitly integrated into the FF) since they are governed by compatible suprathreshold dynamics. However, the resulting model failed to reproduce other subthreshold properties like inward rectification (as observed in the I-V relationships). The oscillatory behavior of the cerebellar GrCs is governed by a slow K^+^ current component (D’Angelo et al., 2001; Gandolfi et al., 2013), while the inward rectification of the subthreshold regime strongly depends on a fast K^+^-dependent component (D’Angelo et al., 2001). Thus, since the AdEx neuron model only includes an additional current component (the adaptation current), we do not have to expect a single set of AdEx parameters fitted to certain spiking properties to also describe both regimes appropriately. Given the computational efficiency but complex adjustment (following a formal analysis) of bursting behaviors of the AdEx model (Brette and Gerstner, 2005), the proposed methodology is presented as a valuable tool to generate a single combination of these few but highly-interrelated parameters for the spiking resonance. The application of this methodology further extending the FF with additional properties from the subthreshold regime would be of interest in helping us to understand how intrinsic properties could affect at the neuron- and also network- level.

The proposed model parameters selected by the EAs (Table 3) are consistent with those equivalent values of biological cerebellar GrCs reported both through the literature and the electrophysiological database (Tripathy et al., 2015; NeuroElectro database, 2019). The resting membrane potential (*E_L_*) in our models are within the experimental range from the electrophysiological database (−73.91 ± 9.46 mV from Storm et al., 1998; Brickley et al., 2001; Cathala et al., 2003; Gall et al., 2003; Goldfarb et al., 2007; Prestori et al., 2008; Osorio et al., 2010; Usowicz and Garden, 2012; NeuroElectro database, 2019) and further bibliography (from −60 to −85 mV in D’Angelo et al., 1995, 2001; Brickley et al., 1996; Armano et al., 2000), and they are closer to the mono-compartmental detailed model’s values (−65 mV in D’Angelo et al., 2001; Masoli et al., 2017). The spike emission (*V_T_*) values are triggered close to the mean value from the database [at around −41.50 ± 6.43 mV (Brickley et al., 2001; Cathala et al., 2003; Goldfarb et al., 2007; Prestori et al., 2008; Usowicz and Garden, 2012; NeuroElectro database, 2019)] and the computational model of D’Angelo et al. (2001), with a spike peak (*V*_peak_) near the experimental evidence [around 20.23 ± 7.04 mV (NeuroElectro database, 2019) from D’Angelo et al., 1998; Osorio et al., 2010; Usowicz and Garden, 2012]. The membrane capacitance (*C*_m_) values appear low as it is notably characterized in a typical GrC (D’Angelo et al., 1995, 2001; Gandolfi et al., 2013) within the range of experimental evidence [3.46 ± 0.82pF (NeuroElectro database, 2019) from D’Angelo et al., 2001; Cathala et al., 2003; Gall et al., 2003; Goldfarb et al., 2007; Prestori et al., 2008; Osorio et al., 2010; Usowicz and Garden, 2012; Gandolfi et al., 2013; Masoli et al., 2017].

It should be noted that the proposed models resulting from EAs with the I-F curve featured in their FFs (*FF2* and *FF4*) show resonance curves in response to sinusoidal current shifted out of the theta band (higher preferred frequencies). Also, the latencies to the first spike remain longer than those experimentally reported, mainly with low stimulation currents. These differences are more severe to the case of the individual from the *FF2*. This fact may indicate an incompatibility of both firing properties (mean frequency under step-current pulses vs. burst frequency resonance under sinusoidal currents) within the simplified AdEx model. Thus, the GrC behavior complexity being beyond the capabilities of these AdEx models with a single parameter configuration (GrCs have different functioning modes).

Based on the analysis of resonance in subthreshold and suprathreshold (spiking) resonance, it seems clear that the preferred frequencies in these two regimes fall in notably different ranges (while spiking resonance tends to fall between 8 and 10 Hz, as driven through the FF in the EA processes, subthreshold preferred frequency peaks about 2 Hz because this regime was not explicitly selected in the FF that drove the parameter tuning). These results may reassert the possibility that the complexity of the spiking resonance in the AdEx model cannot be directly addressed through the analytical adjustment of the parameters. It has to be noted that the subthreshold resonance analysis considers the neuron as the composite of a capacitive current, a passive current, and an adaptive current, neglecting in this way the influence of exponential current of spike firing and the effect of the dynamics of the refractory period. For this reason, the proposed optimization methodology represents a valuable tool to obtain neuron models fitted to complex features. The EA allowed us to tune the two differential equations of the AdEx model according to a complex set of spiking patterns (spiking resonance, regular firing, and delayed firing) under different stimulation protocols (sinusoidal and step current injections).

Realistic modeling based on recent experimental data has provided novel insights on how intrinsic and extrinsic mechanisms interact in other neural systems as the inferior olive (Negrello et al., 2019). According to these results, strong synaptic activity in the awake brain of mice could vanish the functional impact of subthreshold oscillations. Our methodology provides an initial but fundamental tool for the construction of computationally tractable but realistic computational models for future large-scale studies of the functional impact of neuronal resonance in information processing in the GrL. In the particular case of the cerebellar input layer, it remains unclear how spiking resonance (demonstrated *in vitro* in the granule cells and the Golgi cells) interacts in a recurrent inhibitory loop with feed-forward excitation of the Golgi cell. In this sense, theoretical models have addressed information processing in the GrL (Solinas et al., 2010; Garrido et al., 2013), but these models either have not considered neuronal intrinsic resonance or they have neglected the role of the long-term plasticity in the GrC inputs. In addition to this, theoretical models have demonstrated that external oscillatory activity strongly facilitates learning in excitatory synapses (Masquelier et al., 2009) and inhibitory recurrent networks (Garrido et al., 2016). In any case, further experimental data will be required to fit future computational models to address the functional impact of oscillations in GrL operation.

Based on our results, the AdEx model has shown to be a computationally light approach for the close reproduction of the firing patterns reported from cerebellar GrCs. Recent articles in the literature have proposed modified GLIF point-neuron equations (the so-called Extended Generalized Leaky Integrate-and-Fire model) for the reproduction of experimental traces recorded in different cerebellar cells (Geminiani et al., 2018; Casali et al., 2019). That model allowed the direct application of some experimentally testable parameters together with other optimized ones. We, however, propose a methodology based on automatic parameter tuning through an EA-based exploration (all the behavioral target is integrated through FF definition). It has shown to be effective in fitting the model parameters to diverse spiking responses. Therefore, the optimization process is fast, versatile, and able to capture relevant firing features. Contrary to the methodology proposed in Geminiani et al. (2018) where the optimization algorithm fitted the recorded voltage traces, our approach aims to reproduce the firing characteristics (namely, the burst frequency, the firing rate, and the first-spike latency) of the biological neuron.

To sum up, in this study we present an automatic optimization strategy for the development of computationally efficient neuron models that reproduce realistic firing properties under different stimulation protocols. This methodology was applied to the case of the cerebellar GrC. As a result, a simplified GrC model is proposed, suitable for predicting the main suprathreshold dynamics, such as the spiking resonance at the theta range and the linear frequency coding. This contribution serves as an initial step towards a better understanding of the functional implication of the theta-frequency-band resonance for information processing at the cerebellar cortex. This model provides both efficiency and biological plausibility which will facilitate further computational work in the reconstruction of large-scale models of microcircuits to better understand the computational role of the suprathreshold dynamics of the cell on a large scale.

## Data Availability Statement

The datasets generated for this study are available on request to the corresponding author.

## Author Contributions

MM, JG, and ER: study design. MM and MS-L: literature and database search. JG and MM: EAs methodology. MM, JG, and ER: analysis and interpretation of results. MM and JG: writing of the article. All the authors have read and approved the final manuscript. All the results included in this article are part of MM’s Ph.D. thesis.

## Conflict of Interest

The authors declare that the research was conducted in the absence of any commercial or financial relationships that could be construed as a potential conflict of interest.

## Abbreviations

AdEx: Adaptive exponential integrate-and-fire
AP: Action potential
EA: Evolutionary algorithm
FF: Fitness function
GrC: Granule cell
GrL: Granular layer
HH: Hodgkin-and-Huxley
I-F: Intensity-frequency
I-V: Intensity-voltage.
